# Virological failure and associated factors among children receiving anti-retroviral therapy, Northwest Ethiopia

**DOI:** 10.1371/journal.pone.0257204

**Published:** 2021-09-10

**Authors:** Biruk Bayleyegn, Zemene Demelash Kifle, Demeke Geremew

**Affiliations:** 1 Department of Hematology and Immunohematology, School of Biomedical and Laboratory Sciences, College of Medicine and Health Sciences, University of Gondar, Gondar, Ethiopia; 2 Department of Pharmacology, School of Pharmacy, College of Medicine and Health Science, University of Gondar, Gondar, Ethiopia; 3 Department of Immunology and Molecular Biology, School of Biomedical and Laboratory Sciences, College of Medicine and Health Sciences, University of Gondar, Gondar, Ethiopia; Emory University School of Medicine, UNITED STATES

## Abstract

**Background:**

Virological failure is under-recognized issue among children living with human immunodeficiency virus in developing countries. This partly may lead to failure to achieve the global goal of 90-90-90 targets in most developing countries including Ethiopia.

**Objectives:**

This study aimed to assess the virological failure and its predictors among children receiving antiretroviral therapy at the University of Gondar comprehensive specialized hospital, Northwest Ethiopia.

**Methods:**

An institutional based cross-sectional study was conducted among 253 study cohorts from January 2020-April 2021. Socio-demographic characteristics were collected using a structured questionnaire via a face-to-face interview, while detailed clinical data of the children were collected by reviewing the medical record. About 5 ml of blood were collected for the analysis of complete blood count and viral load quantification. Data were analyzed using SPSS version 20 and variables at p-value < 0.05 in the multivariable analysis were considered as statistically significant.

**Results:**

In this study, the viral load suppression rate among antiretroviral therapy experienced children was 68.8%. Meanwhile, the overall virological failure among study participants was 19.4%. Children living without family (AOR = 3.63; 95%CI: 1.27–10.24), children with unemployed family (AOR = 4.95; 95%CI: 1.74–14.12), being wasted (AOR = 3.02; 95%CI: 1.19–7.67) being stunted (AOR = 2.38;95%CI:1.03–5.46), anemia (AOR = 5.50:95%CI;1.37–22.04) and being lymphopenic (AOR = 2.69:95%CI;1.04–7.75) were significantly associated with virological failure among children under treatment.

**Conclusion:**

Higher virological failure among children was noteworthy in the present study. Caretakers other than immediate family, unemployed family, wasted, stunted, anemia, and lymphopenia were significant independent predictors of virological failure. Hence, standard, and optimal management of children under treatment should be warranted.

## Introduction

Pediatric human immunodeficiency virus (HIV) infection is a worldwide public health challenge and mainly affects children living in resource-limited countries. About 1.8 million children are living with HIV in the world and more than 80% of them were living in sub-Saharan Africa [1]. Notably, among 62,194 children under 15 years of age living with HIV, a total of 21,147 children were receiving antiretroviral therapy (ART) in Ethiopia. However, the estimated number of deaths due to HIV was 2900 based on the national estimate of 2017 [2].

The provision of ART has significantly reduced morbidity and mortality in children living with HIV. This was because ART has led to the suppression of HIV-RNA replication, resulting in a dramatic decrease in HIV-RNA viral load. However, virological failure and the development of drug resistance become major challenge in children [3, 4] due to weight-based dosing, poor tolerability of drugs, and suboptimal adherence [5].

Routine plasma HIV-RNA measurement is a more sensitive and early indicator of treatment failure and one of the most essential parameters to predicting disease progression as well as used to decide treatment switch [6]. However, immunological criteria have poor accuracy and are estimated to lead to premature switching to second-line regimens, suggesting immunological criteria is not a good indicator for making decisions about treatment failure [7]. Now a day, the world health organization’s (WHO) recommended that HIV viral load determination is a good marker of therapeutic adherence, disease progression, and treatment efficacy as well as the main therapeutic follow-up parameter rather than CD4 counts [8, 9].

According to WHO, ART treatment failure could be detected either clinically, immunologically, or virologically for the decision to switch either second- or third-line regimens. Virological failure was defined as plasma viral load above 1000 copies/ ml based on two consecutive viral load measurements after 3 months interval, with adherence support. Persistent CD4 levels below 200 cells/mm^3^ for children ≤ 5 years and 100 cells/mm3 for children ≥ 5 years were considered as immunological failure. Moreover, clinical failure was defined as a new or recurrent clinical event indicating the severe or late stage of HIV disease after 6 months of effective treatment [10].

Virological failure and switching to second-line treatment are under-recognized issues among children living with HIV in low- and middle-income countries. Studies in sub-Saharan Africa have shown that the prevalence of virological failure was significantly higher among children compared with adults [11] and was reported 32.1% in Uganda [12], 37% in Kenya [13] and 66% in Malawian children [14].

Ethiopia has adopted the global goal to attain the 90-90-90 targets through monitoring of HIV viral load (at 6 and 12 months after initiating ART and every 12 months thereafter) [2]. Even though the country adopted these guidelines to expanding and strengthening HIV prevention, care, and treatment activities at the national level, only 87% of people living with HIV on ART have attained viral suppression as of May 2018 [15]. Even though shreds of evidences are available on the viral load monitoring in Ethiopia, the pooled prevalence of virological failure in Ethiopia was 5.6% [16] and a study in Amhara region referral hospitals reported that nearly half (48.98%) of the children were developing virological failure [17]. Moreover, few recent studies conducted in different parts of Ethiopia found that high virological failure was detected in children and were reported 28.3% in Bahir Dar [18], 11% in Jimma [19], and 8.3% in Addis Ababa [20]. Accordingly, few studies described virological failure and associated factors with ART outcomes among children in Ethiopia. Thus, in the presence of limited evidence and its clinical importance in the management of children under ART, this study was aimed to assess the virological failure and its predictors among children receiving ART at the University of Gondar comprehensive specialized hospital.

## Materials and methods

### Study population

Institutional based cross-sectional study was conducted among ART experienced children. All children under 15 years of age who attended the clinic for routine visits and took ART treatment for at least 6 months during the study period were eligible for enrolment in the study. Meanwhile, children <6 months on ART treatment, poor blood sample quality and lost to their follow up at the time of data collection were excluded. Based on WHO recommendation, the most prescribed ART regimen for children were consisting of two nucleoside reverse transcriptase inhibitors (NRTIs- lamivudine, abacavir and zidovudine) with a non-nucleoside reverse transcription inhibitor (NNRTI, either nevirapine or efavirenz) or a protease inhibitor (lopinavir). Additionally, the release of a new, dispersible tablet formulation of dolutegravir (DTG) plus two nucleoside reverse transcriptase inhibitors (NRTIs) is now a preferred ART regimen for children with HIV [21].

The study was conducted at the UoGCSH ART clinic from January 2020 to April 2021. The hospital is found in Gondar town and 740 km from Addis Ababa, which is the capital city of Ethiopia. The UoGCSH is the teaching hospital that provides teaching activities to medical and health science students and is the oldest academic institution in Ethiopia. It provides medical, surgical, psychiatric, and many other services to more than 7 million people from Gondar province and neighboring regions. The hospital has both inpatient with more than 512 beds and outpatient services. It also provides HIV/AIDS intervention activities like free diagnosis, treatment, and monitoring in its ART clinic for both pediatrics and adults. The hospital started delivering ART service in 2003 and free charge ART was launched in 2005.

### Definition of variables

Virological failure and viral suppression rate were the outcome variables. While socio-demographic characteristics of children (age, gender, residence, educational status of family, family income, family size, and family occupation), anthropometrical measurement (weight for age, height for age and weight for height), opportunistic infection (OI), ART status and WHO clinical stage were the independent variables. Cytopenia including anemia, leukopenia, and thrombocytopenia were also determinant variables.

#### Virological failure

Plasma viral load >1,000 copies/ml based on two consecutive viral load measurements after 3-months with enhanced adherence support following the first viral load test, after at least six months of starting a new ART regimen. Virologic suppression: HIV plasma viral load <1000 copies/ ml based on one viral load measurement [22, 23].

#### Cytopenia

Was defined as reduction in either of red blood cells, white blood cells or platelets. Anemia: was defined as Hgb concentration of less than 11 g/dl for 6–59 months of age, less than 11.5 g/dl for ages 5–11 years, and less than 12 g/dl for ages 12–14 years old children. Leukopenia: defined as white blood cell count <4000/mm^3^ and lymphopenia: as lymphocyte count <1500/mm^3^ [24, 25].

#### Adherence

Was defined as the degree to which the patient’s behavior is in agreement with the health care provider’s recommendations [26]. It was assessed by pill count and child/caretakers self-report and considered as good adherence (<95%) if the patient miss to take ≤2 of 30 prescribed doses, fair adherence (85–94%) if the patient miss to take 3–5 of 30 prescribed doses, and poor adherence (<85%) if the patient miss to take less than 6 of 30 prescribed doses [27].

### Sample size determination and sampling technique

To recruit study participants who meet the inclusion criteria, non-probable convenient sampling technique was used as they present to the clinic until the calculated minimum sample size was reached. The sample size was calculated using single population proportion formula by considering the prevalence of virological failure, 10.7% [28] using the assumption of 5% margin of error and 95% confidence level (Za/z = 1.96); n = $\frac{\left(Z\frac{a}{2})2\;P(1-P\right)}{d2}$ = 147. After adding 10% non-response rate, the minimum calculated sample size was 162 children living with HIV. Interestingly, the available data was more than a required threshold at the time of the study, finally 253 available study participants were considered for analysis and were included in this study to determine virological failure.

### Data collection procedures

Socio-demographic characteristics of children (such as age, gender, residence, educational status, family income, family size and family occupation) were collected using a pre-tested structured questionnaire via a face-to-face interview technique. Moreover, detailed clinical data of the children such as HIV disease stage, presence of OI, type of ART and duration of ART were collected by reviewing the medical records. Other findings including the history of treatment interruption, the status of adherence to ART regimen and the history of ART regimen change were recorded.

Anthropometric measurement including *Z*-scores of weight-for-age (WAZ), height-for-age (HAZ) and weight for height (WHZ) were calculated using WHO anthro (for children aged ≤5 years) and anthro-plus (for children aged >5 years) soft ware’s based on WHO nutritional assessment guideline [29].

### Laboratory procedures

About 5ml of blood sample was collected following standard operating procedures (SOPs) for complete blood cell count (CBC) and viral load determination. CBC parameters were analyzed using Sysmex KX21 hematology analyzer in accordance with the manufacturer’s instructions by well experienced hematologist. Furthermore, HIV viral load was determined directly by an advanced molecular technique using TAQMAN® AMPLICOR HIV-1 MONITOR (Roche Molecular Systems) according to the manufacturers instruction by well-trained laboratory technologists. For intestinal parasite examination, test tub flotation concentration procedures were done for the detection of protozoan cysts, helminthic ova, and larvae in addition to using the direct wet mount as per the standard protocol.

### Data quality assurance

To assure the quality of data, half day training was given for data abstractors and blood sample collectors before the commencement of data collection, and daily close supervision were made during the data collection period. To maintain consistency, the questionnaire was prepared in English and then translated to Amharic language and then back to English. The questionnaire was pre-tested on 5% of the sample size apart from the actual study area. Then, necessary feedback and modification were done based on its analysis. Safety procedures and specimen handling procedures were strictly followed for all tests (CBC, viral load, and stool examination). Then, manufacturer procedures and SOPs were strictly followed. Moreover, blood sample was checked whether they were in the acceptable criteria like free of hemolysis, no clotting, sufficient volume, correct labeling, and collection time. The performance of automated hematology analyzer was checked by running three levels of hematology controls (normal, low, and high). To check the validity of the automation, negative, low positive and high positive controls were used in each test batch during viral load determination. Furthermore, during post analytical phase the result of all test results (CBC, viral load and stool examination) were registered as the exact number.

### Data analysis and interpretation

Data were coded and entered into EPI-info version 4.4 and were transferred to the statistical package for social science (SPSS) version 20 for analysis. Descriptive statics like frequencies and percentages were used to summarize the data. The distribution of data was assessed through the Shapiro-Wilk test and a p-value >0.05 in the Shapiro-Wilk test was considered as the data were normally distributed. Both crude odds ratio (COR) and adjusted odds ratio (AOR) with the corresponding 95% confidence interval (CI) were calculated to determine the extent to which the risk factors were associated with virological failure. All determinant variables were subjected to bi-variable analysis for calculating COR. To identify the independent explanatory variables of the dependent variable, factors with p< 0.2 at bi-variable analysis were selected and included in multivariable analysis. The model was then built by dropping the most insignificant factor one at a time in a stepwise manner and finally, in the multivariable analysis, variables with p-value < 0.05 were considered as statistically significant.

### Ethical consideration

The study was conducted after ethically approved by the Research and Ethics Review Committee of the School of Biomedical and Laboratory Sciences, College of Medicine and Health Sciences, University of Gondar. Furthermore, a support and permission letter was secured from the UoGCSH administrator. Written informed consent was signed by all parents/caretakers and assent was sought from children >7 years after describing the benefits and the possible risks of the study following the declaration of Helsinki. Participation in the study was purely a voluntarily basis and refusal was possible. The study participants with abnormal findings were linked to the physicians who are working at ART clinic for proper patient care.

## Results

### Socio-demographic characteristics of study participants

A total of 253 ART experienced under 15 years old children were recruited in this study with almost equal distribution of gender (50.2% male Vs 49.8% female). Majority of the children 188 (74.3%) were in the age group between 11–15 years and mean age ± standard deviation (SD) of the study participants was 12.1± 2.8 years. Regarding the HIV status of their family/caretaker, about 213(84.2%) of the study participants were living with HIV positive family and 225 (88.9%) were from urban settings (Table 1).

**Table 1:** Socio-demographic characteristics of ART experienced children and their family /caretaker visiting UoGCSH ART clinic, Northwest Ethiopia, 2021.

Variable	Category	Frequency(N)	Percent (%)
Gender	Male	127	50.2
	Female	126	49.8
Age	2–10	65	25.7
	11–15	188	74.3
Residence	Urban	225	88.9
	Rural	28	11.1
Educational status	No school	21	8.3
	Primary school	201	79.4
	Secondary school	31	12.3
Family income per month (ETB)	≤ 1000	121	47.8
	1001–2000	78	30.8
	>2000	54	21.3
Family size.	<4	181	71.5
	4–6	56	22.1
	>6	16	6.3
Relationship to children	Immediate family	212	83.8
	Caretakers	41	16.2
Family occupation	Employed	168	66.4
	Unemployed	85	33.6
Parental status	Both alive	141	55.7
	Father live	18	7.1
	Mother live	62	24.5
	Both dead	32	12.6
HIV status of caretaker	Positive	213	84.2
	Negative	26	10.3
	Not known	14	5.5
Family education	No formal education	86	34
	Primary school	83	32.8
	Secondary and above	84	33.2

**Note:—**Caretakers = not directly related to the child, ETB = Ethiopian Birr

### Clinical characteristics of study participants

Regarding the clinical presentation, most of the children 243(96%) were under HIV disease stage I (no AIDS case). About 221(87.4%) of the study participants were taking ART for greater than 1 year at the time of the study. Among the study participants, 106 (41.9%) were under DTG containing ART regimen as compared to other NNRTI and protease-inhibitor based regimens. Based on WHO nutritional assessment guideline, nearly half of the children 109 (43.1%) were stunted (Table 2).

**Table 2:** Clinical characteristics and laboratory findings of the study participants attending at UoGCSH, Northwest Ethiopia, 2021.

Variables	Category	Frequency(N)	Percent (%)
WHO stage	I	243	96
	II and above	10	4
WAZ	Wasted	44	17.4
	Normal	109	82.6
HAZ	Stunted	109	43.1
	Normal	144	56.9
OIs	Yes	28	11.1
	No	225	88.9
Adherence	Good	234	92.5
	Fair	14	5.5
	poor	5	2
Duration of ART	6-12month	32	12.6
	>12month	221	87.4
ART treatment interruption	Yes	22	8.7
	No	231	91.3
Current ART types	2NRTI+EFV	57	22.5
	2NRTI+NVP	50	19.8
	2NRTI+LPV/r	40	15.8
	2NRTI+DTG	106	41.9
Intestinal parasite identified on stool microscopy	Yes	57	22.5
	No	196	77.5
Viral load 1	Not detected	109	43.1
	≤ 1000copies/ml	65	25.7
	>1000copies/ml	79	31.2
Viral suppression after a single viral load determination	Suppressed	174	68.8
	Not suppressed	79	31.2
Viral suppression after a second viral load determination	Re-suppressed	30	38
	Virally failed	49	62
Anemia	Yes	54	21.3
	No	199	78.3
Leukopenia	Yes	31	12.3
	No	222	87.7
Lymphopenia	Yes	27	10.7
	No	226	89.3
Cytopenia	Yes	79	31.2
	No	174	68.8

**NB:** WAZ = Weight for Age; HAZ = Height for Age; OIs = Opportunistic Infections; WHO = World Health Organization; ART = Anti-Retroviral Therapy, NRTI = Nucleoside Reverse Transcriptase Inhibitors, EFV = Efavirenz, NVP = Nevirapine, LPV/r = Lopinavir, and DTG = Dolutegravir.

### Laboratory findings and virological failure among children

Viral load suppression rate among children under ART at the UoGCSH ART clinic was 174/253(68.8%) during the first viral load measurement. Then, those study participants whose viral load counts >1000RNA/ml (N = 79) were followed for three months with intensive adherence counseling. After the second viral load assessment, 30/79 (38%) of study participants were virally suppressed whereas the remaining 49/79(62%) of those who were virally not suppressed at the first viral load measurement were not re-suppressed and considered as confirmed virological failure. Therefore, the overall virological failure among ART experienced children was 49/253 (19.4% (95%CI:14–24)) after two times viral load measurement. Regarding hematological profiles’, the prevalence of anima, lymphopenia, and cytopenia were 54(21.3%), 27(10.7%), and 79(31.2%) among ART experienced children, respectively. Moreover, one or more intestinal parasites were detected among 57 (22.5%) of children and 28 (11.1%) of study participants had opportunistic infections (Table 2 above).

### Factors associated with virological failure among children with ART

Both bivariant and multivariant analysis were fitted to determine the associated factors of virological failure. Explanatory variables including gender, relationship to children, family occupation, HIV status of caretaker, WAZ, HAZ, ART types, anemia, lymphopenia and cytopenia were associated with virological failure in bivariant analysis with a p-value <0.2 and become a candidate variable for the final regression model. After adjusting the potential confounders through multivariant logistic regression analysis, children living without family (AOR = 3.63;95%CI:1.27–10.24; P-value = 0.01), children with unemployed family (AOR = 4.95;95%CI:1.74–14.12; P-value = 0.001), being wasted (AOR = 3.02; 95%CI:1.19–7.67;P- value = 0.02), stunted (AOR = 2.38;95%CI:1.03–5.46; P-vale = 0.04), anemia (AOR = 5.5:95%CI;1.37–22.04; P-value = 0.02) and being lymphopenia (AOR = 2.69:95%CI;1.04–7.75; P-value = 0.04) were the independent determinant and significantly associated with virological failure (Table 3).

**Table 3:** Factors associated with virological failure among children on ART attending at UoGCSH ART clinic, Northwest Ethiopia, 2021 (N = 253).

Variable	Category	Virological failure		COR	P-value	AOR	P-value
		No%	Yes%	95%CI		95%CI	
Gender	Male	97(76.4)	30(23.6)	1.74(0.92–3.29)	0.09	1.38(0.62–3.06)	0.44
	Female	107(84.9)	19(15.1)	1		1	
Age	2–10	51(78.5)	14(21.5)	0.83(0.42–1.67)	0.61		
	11–15	153(81.4)	35(18.6)	1			
Family income per month	≤ 1000	97(80.2)	24(19.2)	0.78(0.36–1.68)	0.53	0.7(0.26–1.89)	0.48
	1001–2000	66(84.6)	12(15.4)	0.57(0.24–1.38)	0.21	0.53(0.18–1.6)	0.26
	>2000	41(75.9)	13(24.1)	1		1	
Relationship to children	Immediate family	175(82.5)	37(17.5)	1		1	
	Caretaker	29(70.7)	12(29.3)	1.96(0.92–4.19)	0.08	3.63(1.27–10.24)	**0.01**
Family occupation	Employed	133(79.2)	35(20.8)	1		1	
	Unemployed	71(83.5)	14(16.5)	1.33(0.67–2.64)	0.20	4.95(1.74–14.12)	**0.001**
HIV status of caretaker	Positive	175(82.2)	38(17.8)	0.57(0.26–1.25)	0.16	1.92(0.29–12.82)	0.50
	Negative	29(72.5)	11(27.5)	1		1	
WHO stage	I	197(81.1)	46(18.9)	1			
	II and above	7(70)	3(30)	0.54(0.13–2.19)	0.39		
WAZ	Wasted	31(70.3)	13(29.7)	2.02(0,96–4.23)	0.06	3.02(1.19–7.67)	**0.02**
	Normal	173(82.6)	76(17.2)	1		1	
HAZ	Stunted	81(74.3)	28(25.7)	2.02(1.08–3.81)	0.03	2.38(1.03–5.46)	**0.04**
	Normal	123(85.4)	21(14.6)	1			
OIs	Yes	21(75)	7(25)	1.45 (0.58–3.64	0.42		
	No	183(81.3)	42(18.7)	1			
Duration of ART	6-12month	26(81.2)	6(18.8)	1			
	>12month	178(80.5)	43(19.5)	1.05(0.41–2.70)	0.92		
ART treatment interruption	Yes	16(72.7)	6(27.3)	1			
	No	188(81.4)	43(18.6)	0.61(0.23–1.65)	0.33		
Intestinal parasite	Yes	44(77.2)	13(22.8)	1.31(0.64–2.69)	0.46		
	No	160(81.6)	36(18.4)	1			
Anemia	No	172(86.4)	27(13.6)	1		1	
	Yes	32(59.3)	22(40.7)	4.38(2.22–8.62)	0.00	5.50(1.37–22.04)	**0.02**
Leukopenia	No	180(81.1)	42(18.9)	1			
	Yes	24(77.4)	7(22.6)	1.25(0.50–3.09)	0.63		
Lymphopenia	No	187	39	1		1	
	Yes	17	10	2.82(1.20–6.62)	0.17	2.69(1.04–7.75)	**0.04**
Cytopenia	No	152(87.4)	22(12.6)	1		1	
	Yes	52(65.8)	27(34.2)	3.59(1.88–6.84)	0.00	0.57(0.18–2.48)	0.56

COR = crude odds ratio, AOR = adjusted odds ratio, CI = confidence interval, WAZ = Weight for Age, HAZ = Height for Age, OIs = Opportunistic Infections, WHO = World Health Organization, ART = Anti-Retroviral Therapy. Bold numerals were indicating significantly associated variables.

## Discussion

Virological failure is the gold standard method to detect HIV treatment failure [8]. Accordingly, in this study the overall virological failure among ART experienced children was 19.4% (95% CI:14–24) after two consecutive viral load measurement and the viral load suppression rate was 68.8% during the first viral load measurement at the time of the study.

The prevalence of virological failure among children in this study was in line with a study conducted in Bahir-Dar 14.8% [30] and southern Ethiopia 15% [31]. Whereas, the prevalence reported in this study was far higher than the previous study conducted in different segment of the country, 8.3% in Tikur Anbessa Specialized Hospital, Ethiopia [20], 10.5% in Bahir-Dar, Ethiopia [28] and 11% in Jimma, Ethiopia [19]. As compared to other studies conducted abroad, virological failure in this study is lower than the prevalence reported in India 29% [32], in Kenya 37% [13], in Senegal 64% [33] and 66% in Malawian children [14]. This high prevalence of virological failure among ART experienced children in this study might be probably due to sample size variation and differences in study design and study population (inclusion and exclusion criteria).

Anti-retroviral therapy experienced children living with caretakers had four times higher risk of developing virological failure relative to their counterparts, children living with their immediate family (AOR = 3.63: 95%CI; 1.27–10.24). This was evidenced by different established studies conducted in Ethiopia, children not having a family as a primary caretaker were more prone to treatment failure [20, 22]. This might be explained by caretakers other than immediate family may give far less attention to children and become psychologically depressed. Yet ART experienced children living with family members are highly motivated to ensure treatment success.

Moreover, this study also showed that ART experienced children with unemployed family were 5 times more likely to have a higher risk of getting virological failure (AOR = 4.95; 95%CI:1.74–14.12) compared to those who have employed family. This is similar to a study conducted by Owusu.M *et al* [34]. Unemployed family reflect deprived socio-economic condition which does not achieve adequate dietary intake and healthy nutrition to their children, which may play a central role for the acceleration of viral replication through immune dysfunction.

This study also revealed that the risk of virological failure among wasted study participants was 3 times more likely greater than that of the normal study participants (AOR = 3.02: 95%;1.19–7.67). This is not in agreement with the study conducted among children in South Africa [35], the WHO WAZ value<-3 is not independently associated with virological failure. This difference is probably attributed to sample size variation (253 versus 5485) and study design (cross-sectional versus prospective).

Moreover, stunted study participants had significantly higher odds of getting virological failure (AOR = 2.38; 95%CI:1.03–5.46) as compared to children with normal nutritional status in this study. This is in line with a study conducted in western Kenya [13] stated that moderate and severe malnutrition is a risk factor of virological failure among children. Another similar study conducted in Ethiopia also showed that patients with severe malnutrition were found at high risk of getting virological failure [36]. This is partly because of that food insecurity (malnutrition) can impair the immune system and contribute to the progression of HIV disease even they were under ART treatment [37].

Anemia is one of the significant independent factors for virological failure among children living with HIV in this study. Accordingly, anemic children were nearly six times more likely to have a higher risk of developing virological failure when compared with their counterparts, non- anemic children (AOR = 5.5; 95%CI: 1.37–22.04). This has concurred with a comparative study conducted by Ruhinda N *et al* reported that non- anemic children achieved a higher mean of reduction in viral load and significantly higher proportion attained complete viral suppression as compared to anemic children [38]. Cytopenia including anemia increases as the disease progress to AIDS stage and it increases the probability of death in children [24, 39, 40].

In the present study, the odds of getting the risk of virological failure among children with low lymphocyte count (lymphopenia) were three times (AOR = 2.69; 95%CI: 1.04–7.75) higher than that of non-lymphopenia children. In fact, a number of previous studies illustrated that total lymphocyte count is a potential surrogate marker of immune function among patients living with HIV [41–43]. Hence, once the children’s lymphocyte count become depleted, the robustness and functionality of the immune system to protect the body against OI is distorted and gives a hospitable environment for the multiplication of HIV RNA.

### Limitation of the study

The main limitation of this study was unable to perform drug resistance due to unavailability of the test in the country. The other limitation of the present study was the enrollment of small sample size and single centered nature of the study which lacks generalizability.

## Conclusion

In this study, higher virological failure and lower viral suppression rate was evidenced among ART experienced children. Caretaker other than immediate family, unemployed family/caretaker, wasted, stunted, anemia and lymphopenia were a significant independent predictor of virological failure among ART experienced children. Hence, governmental, and non-governmental organization should invest their effort to ensure optimal management of those vulnerable children under treatment. Furthermore, longitudinal, and large sample size research including drug resistance is encouraged.

## Abbreviation/Acronym

AIDS: Acquired Immunodeficiency Syndrome
CBC: Complete Blood Cell Count
ART: Antiretroviral Therapy
HAZ: Height-for-Age
HIV: Human Immunodeficiency Virus
SOP: Standard Operating Procedures
UoGCSH: University of Gondar Comprehensive Specialized Hospital
WAZ: Weight -for-Age
WHO: World Health Organization
WHZ: Weight-for-Height
